# Different gastric tubes in esophageal reconstruction during esophagectomy

**DOI:** 10.1007/s10388-023-01021-z

**Published:** 2023-07-25

**Authors:** Shaowu Sun, Zhulin Wang, Chunyao Huang, Kaiyuan Li, Xu Liu, Wenbo Fan, Guoqing Zhang, Xiangnan Li

**Affiliations:** Department of Thoracic Surgery, First Affiliated Hospital of Zhengzhou University, No. 1 Jian She Road, Zhengzhou, 450052 Henan Province China

**Keywords:** Esophagectomy, Esophageal reconstruction, Gastric tube

## Abstract

Esophagectomy is currently the mainstay of treatment for resectable esophageal carcinoma. Gastric grafts are the first substitutes in esophageal reconstruction. According to the different tailoring methods applied to the stomach, gastric grafts can be classified as whole stomach, subtotal stomach and gastric tube. Gastric-tube placement has been proven to be the preferred method, with advantages in terms of postoperative complications and long-term survival. In recent years, several novel methods involving special-shaped gastric tubes have been proposed, which have further decreased the incidence of perioperative complications. This article will review the progress and clinical application status of different types of gastric grafts from the perspectives of preparation methods, studies of anatomy and perioperative outcomes, existing problems and future outlook.

## Introduction

Esophageal cancer is one of the most aggressive cancers of the digestive system. It is the 7th most common cancer and the 6th most common cause of mortality worldwide [1]. Despite advances in diagnostic and therapeutic strategies, the overall 5-year survival rate for patients with esophageal cancer is still 15% to 20% worldwide [2]. Because the early symptoms of esophageal cancer are hidden and of low specificity and because routine gastroscopy is not yet widespread, many patients are diagnosed as having middle advanced-stage cancer at the time of initial diagnosis [3]. Therefore, surgery plays an important role in the treatment of esophageal cancer. Curative esophagectomy is the cornerstone treatment for locally or locally advanced esophageal cancer [4].

Surgical treatment of esophageal cancer has a history of more than 200 years. In the early twentieth century, skin, jejunum, colon, stomach, and artificial materials were used as esophageal substitutes. By the 1950s, the colon and stomach had become the most commonly used organs for reconstruction [5]. However, because of the technical complexity, many anastomoses, and high rates of complication and mortality in the short term after surgery, esophageal replacement with colon has not been commonly used after the 1980s [6]. Esophageal reconstruction with the stomach has gradually become the mainstream method. Only if the stomach is unavailable (e.g., tumor invasion, gastrectomy) or if the gastric graft is malfunctioning will colon transplantation be considered [7]. Haverkamp et al. [8] surveyed the preferred method of reconstruction among 435 thoracic surgeons worldwide and found that most of them selected gastric tubes (95%). The jejunal interposition (3%), colonic interposition (2%) and whole stomach (1%) methods were less used.

To date, there are various types of gastric grafts for esophageal reconstruction, such as the whole stomach, subtotal stomach, typical gastric tube and special-shaped gastric tube. However, few studies have focused on the comprehensive analysis and comparison of different types of gastric grafts, especially novel, specially shaped gastric tubes. This article will review the clinical application status and research progress regarding different types of gastric grafts from the perspectives of preparation methods, studies of anatomy and perioperative outcomes, existing problems and future outlook.

## Whole stomach and subtotal stomach

**Fig. 1:**
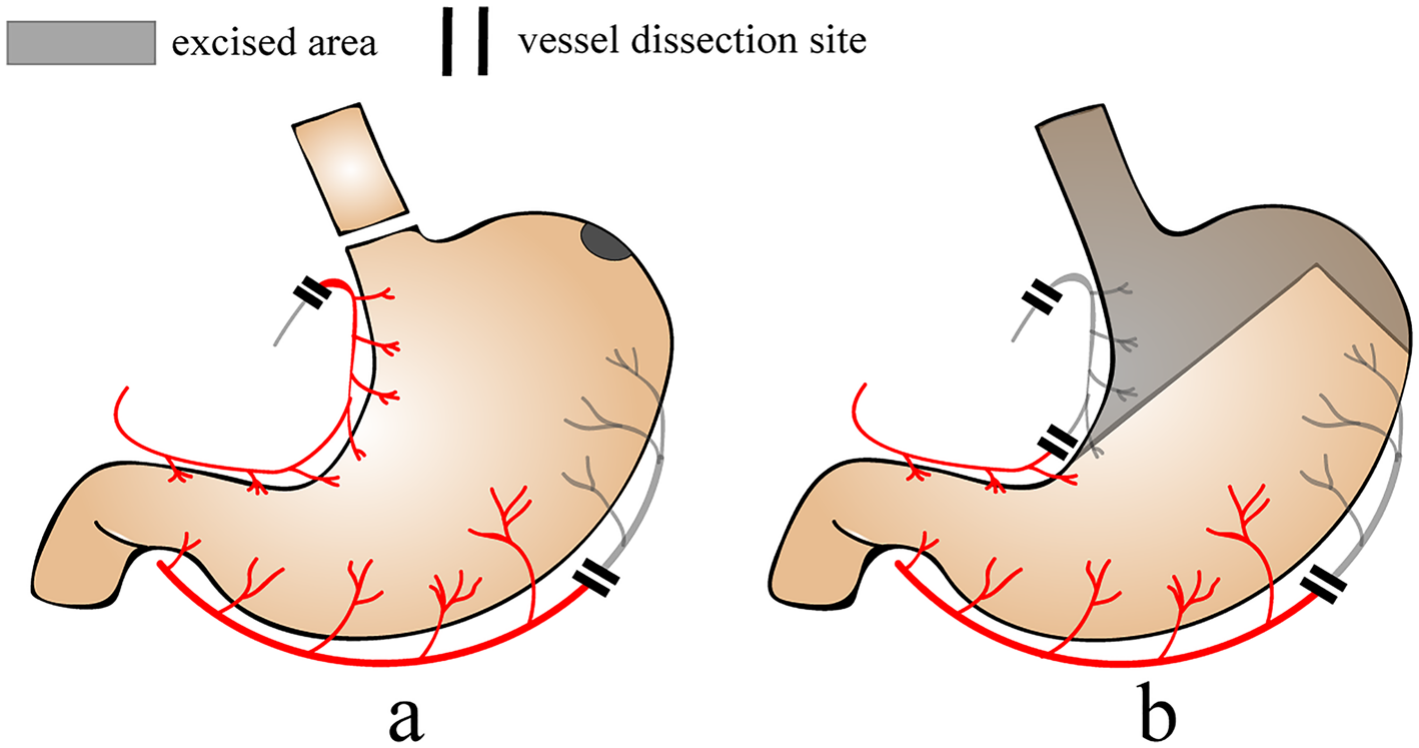
Preparation methods of **a** the whole stomach and **b** the subtotal stomach

Esophageal reconstruction with whole stomach was first described by Kirschner and refined by Akiyama et al. [9]. The left gastric artery and short gastric artery are severed, and the right gastric artery and right gastroepiploic artery are preserved. The esophagus is cut at the gastroesophageal junction, and the incision at the cardia is embedded by a seromuscular suture. The scheduled esophagogastric anastomosis is at the fundus of the stomach (Fig. 1a). The whole-stomach approach maximizes the preservation of the function and vessels of the stomach. However, the whole-stomach approach, because of the lack of length, is less commonly used in esophagectomy for cervical esophageal cancer or hypopharyngeal cancer. According to the radical principle and the rule of lymphatic metastasis of esophageal carcinoma, when the primary tumor is thoracic esophageal carcinoma, the cardia, part of the lesser curvature and peripheral lymph nodes must be removed, which is the preparation method of the subtotal-stomach approach [9] (Fig. 1b). Because of the resection of the lesser curvature, the length of the subtotal stomach is sufficient for anastomosis at the cervical level.

There are many problems with using the whole/subtotal stomach as an esophageal substitute. Due to the large capacity and receptive relaxation of the stomach, grafts in the chest cavity tend to prolapse so that the position and pressure relationship between the stomach and pylorus becomes abnormal, which will cause gastric dilatation, reflux and emptying disorders. The dilated stomach will also compress the heart and lungs, affecting cardiopulmonary function and reducing the quality of life and long-term prognosis of patients [10]. With the prevalence of gastric tubes, the whole/subtotal-stomach approaches are currently less used.

## Typical gastric tube

Gastric tubes, in the early stage, were mainly used when other esophageal substitutes (e.g., whole stomach, colon) did not have enough length for anastomosis. With a deepening understanding, doctors have found that gastric tubes can alleviate postoperative complications such as gastric emptying disorder and thoracogastric syndrome. In recent years, there has been considerable research on gastric tubes, and esophageal reconstruction with gastric tubes has become the first choice of most surgeons [8].

### Preparation method of gastric tube

**Fig. 2:**
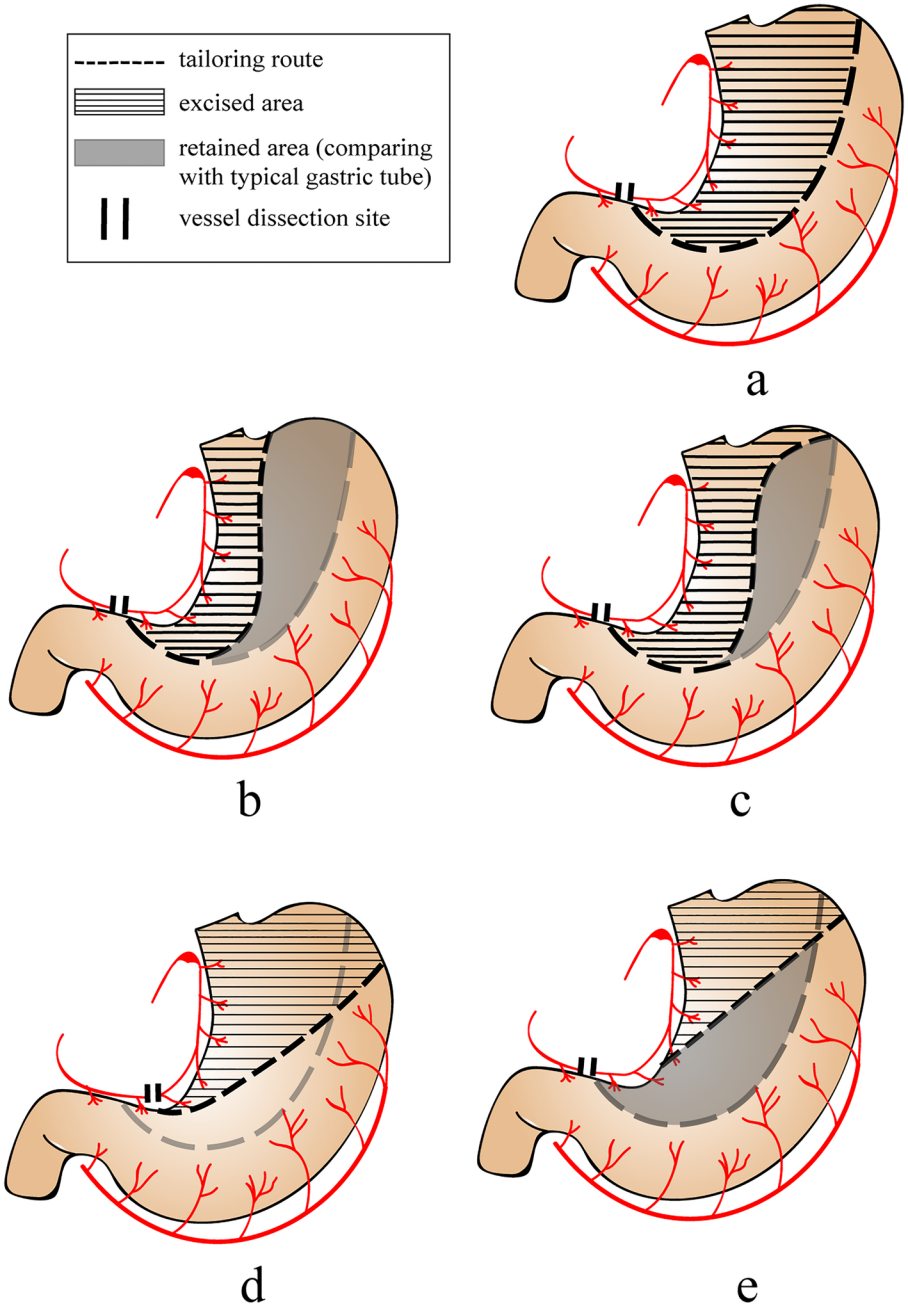
Preparation methods of different gastric tubes **a** Typical gastric tube. **b** Stretched gastric tube. **c** Flexible gastric tube. **d** Coniform gastric tube. **e** Fusiform gastric tube

Gastric tube refers to the tailored lesser curvature and cardia of the stomach along the route parallel to the greater curvature after stomach mobilization, with the width of the gastric tube usually varying from 3 to 6 cm. During mobilization of the stomach, the left gastric artery, short gastric artery and left gastroepiploic artery are severed (Fig. 2a).

### Anatomical studies of gastric tubes

Liebermann et al. [11] found that the nutrition of the gastric tube depends entirely on the right gastroepiploic artery, and this has been recognized by the medical community. Theoretically, the gastric tube, compared with the whole stomach, was supposed to have a worse blood supply due to resection of the lesser curvature. However, Tabira et al. [12] measured the tissue blood flow at the anastomotic site and found that there was no difference in tissue blood flow between the subtotal stomach and the 3 cm gastric tube groups. Sugimachi K et al. [13] and Park SY et al .[14] suggest that the blood supply region of the right gastroepiploic artery decreases because of the resection of the lesser curvature, allowing a greater supply of blood to the cranial part of the gastric tube.

To further extend the length of the gastric tube and alleviate postoperative complications, surgeons created a much narrower gastric tube. However, there is still controversy about the blood supply of the narrow tube. Nodye et al. [15] concluded from 39 cadaver stomach arteriographies that 5 cm gastric tubes were the most ideal, and a narrow gastric tube (4 cm) affected the perfusion of the fundus. Pierie et al. [16] came to similar conclusions. However, as mentioned earlier, the blood supply of 3 cm gastric tubes was not different from that of subtotal stomachs [12]. An animal experiment by Sugimachi et al. [13] in 1982 also showed that there was no statistically significant difference between the whole stomach and gastric tubes larger than 3 cm, but the blood supply of the 1.5 cm gastric tube decreased significantly. According to current research, 3 ~ 6 cm gastric tubes are all available.

In addition, with the application of novel blood flow detection techniques, such as laser Doppler flowmetry (LDF), near-infrared spectroscopy (NIRS), laser speckle contrast imaging (LSCI), fluorescence imaging (FI), and sidestream darkfield microscopy (SDF), the submucosal blood supply can be visualized and quantified, which is especially helpful in studying the blood supply to the anastomosis [17]. Hong et al. [18] conducted a meta-analysis to evaluate the safety and efficacy of using indocyanine green (ICG) fluorescence to determine a suitable anastomotic position during esophagectomy. They conclude that the application of ICG fluorescence before and after gastric management can better prevent anastomosis leakage.

### Postoperative complications, quality of life (QOL), and survival studies of gastric tube placement

#### Anastomosis leakage (AL)

AL is one of the most serious postoperative complications of esophagectomy; it prolongs hospital stay, increases morbidity, reduces the quality of life of patients, and affects long-term survival [19]. The tension and blood supply at the anastomotic site, which are closely related to the method of reconstruction, are the major factors contributing to AL [20]. With the application of various optical techniques for perfusion monitoring, qualitative and quantitative detection of anastomotic blood supply is available [17]. Among the techniques, fluorescence imaging is the most commonly used due to its easy operation, untouched measurement and wide visual field [21].

In general, whether gastric tubes can reduce the incidence of AL remains controversial. Many large-volume studies have shown that gastric tubes can significantly reduce the incidence of AL compared with the whole-stomach approach [22, 23]. However, Zhang et al. [24] conducted a meta-analysis and found no significant difference in the AL incidence between the whole-stomach and gastric-tube groups. Yoshida et al. [25] reported an AL incidence <1% in a study including 300 patients who underwent subtotal-stomach reconstruction. This may be because the occurrence of AL involves several factors, such as the type of gastric tube, anastomosis method and reconstruction route [26]. The anastomosis method can be divided into two types: manual and mechanical anastomosis. Recent reports on the anastomosis method have not reached a consistent conclusion. Therefore, many researchers believe that the anastomosis method is not an important factor after the learning curve [27]. Moreover, esophageal reconstruction can be performed via the anterothoracic, retrosternal, or posterior mediastinal route. At present, the latter two are more commonly used. In terms of the incidence of AL, the posterior mediastinal route may be a better choice [28]. Therefore, more high-quality randomized controlled trial (RCT) studies are needed to further guide clinical application strategies.

#### Pulmonary complications

Pulmonary complications are the most common complications following esophagectomy and have been implicated in nearly two-thirds of postoperative mortalities [29]. Lung infections and atelectasis are the most common occurrences and are the foundation of other pulmonary complications. The esophagectomy affects the function of respiratory muscles, especially the diaphragm. Studies have shown that low tidal volume ventilation for one hour may lead to mild atelectasis [30]. Yamamoto et al. [31] compared postoperative patients with or without pulmonary complications and found that overall survival (OS) was worse in the group with pulmonary complications.

Studies have shown that compared with the volume of the whole stomach, the volume of gastric tubes is reduced by 21%~47% [32]. Theoretically, a tailored stomach in the thoracic cavity has less of an effect on thoracic organs, which allows the lungs to expand sufficiently and ensures blood oxygen exchange, thereby reducing the incidence of pulmonary complications such as breathing difficulties and lung infections [33]; however, there is currently little evidence supporting this theory. Studies by Zhang et al. [34] and Zhang et al. [24] have shown that there is no significant difference in the incidence of pulmonary complications, such as pneumonia and lung impairment, between patients with a narrow gastric tube and a whole stomach. More large-sample controlled trials are needed to confirm the advantages of gastric tubes in terms of respiratory complications.

#### Reflux esophagitis

Reflux esophagitis is one of the most annoying complications following esophagectomy and can significantly reduce the quality of life by leading to emesis, night aspiration, weight loss and anastomotic leakage and stricture. On the one hand, esophagectomy destroys the structural basis of antireflux, including the lower esophageal sphincter, angle of His, diaphragmatic crus, and phrenoesophageal ligament. On the other hand, gastric retention caused by delayed thoracic stomach emptying can also worsen reflux. Many studies have shown that the emptying function of gastric tubes is faster, and therefore, gastric tubes are associated with a lower incidence of reflux [35].

Resection of the lesser curvature reduces the number of chief cells and parietal cells, resulting in decreased peptic acid secretion in the gastric tube. Vagotomy and reduction of gastric submucosal blood supply also contribute to this. All these factors lead to a low chance of reflux [36]. A prospective, randomized study of 10-year follow-up showed that patients in the narrow-gastric-tube group had significantly less postoperative reflux and better prognosis than those in the whole-stomach group [34]. Almost all other studies support this conclusion [24, 37].

#### Long-term survival rate

The survival rate after esophagectomy is related to many factors, including patients’ nutritional status, minimally invasive surgery, anastomosis method and location, the number of dissected lymph nodes, tumor stage and incidence of postoperative complications. Different methods of esophageal reconstruction affect the long-term survival rate mainly effects related to the number of dissected lymph nodes, postoperative complications and anastomotic blood supply.

Zhang et al. [38] found that the rates of recurrence or metastasis in the gastric-tube group were lower than those in the whole-stomach group 1 and 2 years after surgery, and the survival rate of the gastric tube group was significantly better (80% versus 61%). They suggest that abdominal lymph node metastases from thoracic esophageal cancer usually involve lesser curvature. Resection of the cardia and lesser curvature in the gastric tube reduces the recurrence of carcinoma.

Postoperative complications have a great impact on postoperative quality of life and the long-term prognosis of tumor diseases. Booka et al. [39] conducted a meta-analysis including 21 studies on postoperative complications of esophageal cancer and found that pulmonary complications and anastomotic leakage were associated with a significant reduction in overall 5-year survival. Zhang et al. [34] conducted a prospective, randomized study involving a 10-year follow-up to compare the prognosis between the gastric tube and whole-stomach groups. They found that the 1-year, 2-year, 5-year and 10-year survival rates and the probability of metastasis and recurrence in the gastric-tube group were better than those in the whole-stomach group. Therefore, gastric tube reconstruction may be a much better choice.

#### Quality of Life (QOL)

Health-related QOL has been advocated by the U.S. FDA as the second-most relevant outcome measure for assessing cancer therapy [40]. When evaluating the effect of treatment on highly malignant tumors associated with major surgical trauma, attention should be paid not only to the long-term survival rate but also to the QOL of patients after surgery. However, there are few studies comparing the QOL of patients after esophagectomy [41].

To date, there has been only one systematic study concerning the QOL comparison between patients with whole stomach reconstruction and those with gastric tube reconstruction, which lasted more than 10 years. In the study, the researchers found that patients with gastric tube reconstruction had better QOL scores in the early-term follow-up (< 1 year), especially in terms of gastroesophageal reflux, dysphagia and dyspnea. However, QOL differences were resolved in the long-term follow-up. Among patients who survive 10 years after esophagectomy, there was no significant difference in QOL scores between the two reconstruction methods except for worse dysphagia scores among patients with whole stomach reconstruction [22, 34, 37].

Therefore, whole stomach and gastric tube reconstruction are both feasible choices in terms of the QOL of patients in the long term. However, the persistence of dysphagia symptoms may be correlated with worse survival among patients with whole stomach reconstruction. More large-scale controlled trials are needed to explore reconstruction method selection based on QOL scores.

## Special-shaped gastric tube

The innovation of the gastric-tube approach is based on three main points: blood supply, tension, and storage function. The main goal in the evolution of gastric tube preparation methods is reduction of postoperative complications, especially anastomotic leakage, so most innovations revolve around the blood supply and tension of gastric tubes. In addition to the typical gastric tube, the special-shaped gastric tube is also under constant exploration. In the second half of the last century, surgeons developed various special-shaped gastric tubes—reversed gastric tubes, isoperistaltic gastric tubes, elongated gastric tubes and fundus rotation gastric tubes—but these are not widely used in clinical practice.

In recent years, various preparation methods of special-shaped gastric tubes have been proposed and the advantages of typical gastric tubes are well combined in all of them. They are more in line with the physiological characteristics of the normal digestive tract and have a better anastomotic blood supply, significantly reducing the incidence of postoperative complications (Table 1). Compared with typical gastric tubes, special-shaped gastric tubes have many advantages.

**Table 1:** Studies published on special-shaped gastric tubes

Type	Reconstruction route	Anastomosis method	Anastomotic leakage	Pulmonary complications	Severe reflux
Stretched gastric tube [42]	PM	Handsewn	7.6%	–	–
Baseball bat-like gastric tube [43]	PM	Handsewn	7.3%	6.7%	10.5%
Flexible gastric tube [44]	RS	Stapled	1.8%	–	–
Coniform gastric tube [45]	PM	Handsewn	2.5%	7.4%	9.8%
Fusiform gastric tube [46]	PM	Stapled	0	7.8%	–

– not mentioned, *PM* posterior mediastinal route, *RS* retrosternal route

### Stretched gastric tube and baseball bat-like gastric tube

Miyawaki et al. [42] first reported the preparation method for stretched gastric tubes in 2020. After mobilization of the stomach, the right gastric artery is dissected on the peripheral side, and resection of the lesser curvature begins from the stomach antrum. The tailored route of the lower segment is the same as in the method of preparing a typical 3 cm gastric tube, the objective of which is to maintain a sufficient length of the total gastric tube. From the middle of the gastric tube, the width increases along the rest of the way up to the end for the purpose of maintaining blood flow in the anastomotic region (Fig. 2b).

This method was used to conduct esophageal reconstruction for 67 patients following esophagectomy. Compared with 121 patients who underwent 3 cm narrow-gastric-tube reconstruction in the early stage, the incidence of anastomotic leakage was significantly reduced in patients who underwent stretched-gastric-tube reconstruction. Multivariate analysis showed that the shape of the gastric tube was an independent risk factor for anastomotic leakage, but there was no clear relationship to other postoperative complications.

Lai et al. [43] also reported a similar method in 2022, which is called the baseball bat-like gastric tube. In this study, they compared 613 patients from the center who underwent narrow-gastric-tube reconstruction or baseball bat-like gastric-tube reconstruction at the same time using the propensity score-matching method. They found that the incidence of anastomotic leakage was significantly lower in the baseball bat-like gastric-tube group (7.5% versus 14.2%), and there were no significant differences in other major complications between the two groups.

### Flexible gastric tube

Nakajima et al. [44] first reported the flexible gastric tube preparation method in 2020. Similar to the 4 cm narrow-gastric-tube method, the resection of the lesser curvature was started at approximately 5 cm proximal to the pylorus along the route parallel to the greater curvature. At 3 to 5 cm proximal to the final branch inflow portion of the right gastroepiploic artery, the cutting line was turned to the lesser curvature and toward the tip of the gastric tube (Fig. 2c).

The authors believe that the construction of a flexible-tube stomach combines the advantages of a narrow gastric tube and a subtotal stomach, increases the blood supply of the fundus, and thus reduces the incidence of anastomotic leakage. At the stomach antrum, more gastric tissue is removed by the narrow-gastric-tube method to reduce the shunting of the right gastroepiploic artery. Subsequently, the upper segment of the flexible gastric tube is tailored by the subtotal-stomach method, maximizing the preservation of the vascular plexus in the upper gastric wall. This allows more blood flow from the right gastroepiploic artery to pass through the vascular plexus in the upper wall to nourish the tip of the gastric tube, thereby maintaining more blood flow at the anastomosis.

Since 2000, this center has operated on 615 patients with esophageal cancer using this method, with only 11 patients developing anastomotic leaks (1.8%) and one patient developing gastric tube necrosis, which is much better than outcomes with a typical gastric tube.

### Coniform gastric tube

Zheng et al. [45] first reported the preparation technique of a coniform gastric tube in 2019. The resection of the lesser curvature was started at approximately 2 ~ 3 cm proximal to the pylorus and then tailored toward the greater curvature of the fundus along the superior border of the gastric body. The overview of the gastric tube showed a coniform shape that was narrow at the top and wide at the bottom (Fig. 2d).

They believe that the coniform gastric tube combines the advantages of a wide and narrow gastric tube. First, the resection of the fundus with poor blood flow improves the blood supply of the whole gastric tube. Second, the upper segment of the coniform gastric tube is narrower, which is less prone to thoracogastric dilatation and has less effect on lung function. Third, the lower segment of the coniform gastric tube is wider, which allows more food storage and improves the postoperative feeding experience of patients. Finally, the end-to-end anastomosis could fully utilize the length of the gastric tube without an additional gastric stump. Since 2016, the incidence of anastomotic leakage and thoracogastric dilation has been 2.5% and 4.9%, respectively, among the 122 patients who have been treated with this method, both better than observed among patients treated with the typical gastric-tube reconstruction performed at the center.

### Fusiform gastric tube

Yuan et al. [46] first reported the fusiform gastric-tube preparation method in 2018. The number of preserved right gastric artery branches is decided according to the principle of symmetrically equal length and tension in the lesser and greater curvature. The final branch inflow portion of the right gastric artery is the tailoring endpoint. A line is drawn between the endpoint and the tip of the junction of the lesser and greater vessel arcades. The intersection between this line’s reverse extension and the greater curvature is the starting point. The line between the starting point and the endpoint is the route of resection. The overview of the gastric tube reveals a fusiform shape that is wide in the middle and narrow on both sides (Fig. 2e).

The authors believe that the fusiform gastric tube retains parts of the branches of the right gastric artery and reduces the shunting in the right gastroepiploic artery at the side of lesser curvature, ensuring sufficient blood supply to the gastric graft. In addition, the fusiform gastric tube artificially creates an enlarged area in the middle of the graft, which alleviates compression to the respiratory and digestive systems and, thus, reduces the incidence of postoperative complications.

This study compared the intraoperative blood flow detection and postoperative complications between the narrow-gastric-tube group (45 patients) and the fusiform-gastric-tube group (51 patients). They found that blood flow in the antrum, body and bottom of the stomach in patients in the fusiform-gastric-tube group was better than corresponding blood flow in patients in the narrow-gastric-tube group; there were also fewer pulmonary complications and less postoperative anastomotic leakage.

## Future outlook

### Integrated strategies (ISs) for esophagectomy involving improved gastric tube

Simply optimizing gastric tubes sometimes does not yield significant benefits. The postoperative outcomes of patients are affected by a combination of factors, including the reconstruction method, anastomotic method, minimally invasive operation, microvascular anastomosis, and surgeon’s experience. Ischemic preconditioning [47] and pyloroplasty [48, 49] also have an impact and are under discussion. Making improvements unilaterally can hardly have a definite impact on the outcome. More careful exploration and verification must be applied to achieve simultaneous innovation in many aspects. It is neither practical nor safe for surgeons to choose between different methods subjectively and instantly during surgery. There is an urgent need for integrated strategies (ISs) and technique standardization to ensure the clinical efficacy of esophagectomy.

While optimizing the gastric tube type, attention should be paid to the position of the anastomosis and the preservation of blood vessels to ensure an adequate blood supply. Our center introduced an integrated strategy (IS) in esophageal reconstruction to reduce postoperative complications, especially anastomotic leakage. Three innovations were implemented in this strategy—application of an esophagus-diameter-approximated slender gastric tube, preservation of the fibrous tissue (microvessels) around the residual esophagus and anastomosis at the inferior pole of the thyroid. We emphasized the importance of the blood supply of the residual esophagus to the anastomotic blood supply, which is not covered in previous articles. Our experimental results confirmed that more than 80% of patients’ gastric tube blood supply was dominated by the residual esophagus, and we demonstrated a strikingly lower incidence of anastomotic leakage and a relatively lower incidence of postoperative complications, such as gastric tube dilation and delayed gastric emptying. Additionally, further studies are needed to establish this IS as the standard of care.

### Gastric tube reconstruction in the era of minimally invasive esophagectomy (MIE) and enhanced recovery after surgery (ERAS)

In the past few years, MIE has been shown to be superior to open esophagectomy regarding postoperative outcomes, especially pulmonary complications, without compromising DFS or OS [50]. Moreover, MIE has obvious advantages in terms of surgical trauma, operation time and postoperative recovery. Tsujimoto et al. [51] found that compared to open gastric tube reconstruction, laparoscopy-assisted gastric tube reconstruction significantly attenuates postoperative systemic inflammatory response syndrome, which is associated with frequent postoperative complications. Investigators have reported several new MIE methods, such as robot-assisted MIE [52], mediastinoscopic esophagectomy [53] and flexible gastroscopic esophagectomy [54], which will further reduce surgical trauma and the incidence of postoperative complications. In the future, further studies will be required to confirm the efficacy of these methods.

ERAS can improve perioperative care, minimize complications, and accelerate the recovery of patients. Based on studies of early oral feeding after esophagectomy, Li Y proposed the NTNF (none tube no fasting)-ERAS pattern, involving no nasogastric/nasointestinal/jejunostomy tube, no thoracic/abdominal/cervical drainage tube and no fasting [55], to significantly improve patients’ postoperative nutritional status, clinical outcomes and long-term prognosis. However, the absence of a nasogastric tube may lead to delayed gastric emptying, resulting in gastric tube dilatation and respiratory aspiration. It is important to select the proper type of gastric tube to reduce the risk of food accumulation. Considered along with the above-mentioned results, it seems that narrow-gastric-tube and coniform-gastric-tube reconstruction may be better choices than others.

### Artificial esophagus

There has been great progress in recent years in artificial esophagus research. The use of an artificial esophageal replacement can reduce surgical trauma, simplify surgical procedures, and reduce the impact on digestive function. Liang et al. [56] implanted an artificial esophagus made of nitinol into pigs, which gradually transformed into a lumen covered with a multilayered squamous epithelium. Chung et al. [57] used an artificial esophagus consisting of three-layered poly (ɛ-caprolactone) nanofibers and silk fibroin, providing temporary support for the regenerative process of native tissues in a rat model. Although the use of regenerative tissue as an artificial esophagus may be possible, there is no solution for the missing digestive function of the artificial esophagus. There is still a long way to go before the real esophagus can be replaced with an artificial esophagus.

## Conclusion

The stomach is the preferred esophageal substitute following esophagectomy. The gastric-tube approach is the most commonly used method of esophageal reconstruction, and a width of 3 ~ 6 cm is feasible. The special-shaped gastric tube is an optimized version of the typical gastric tube that may have advantages with respect to the anastomotic blood supply and the incidence of postoperative complications. In the future, screening high-risk patients before and during surgery and the application of integrated strategies, ERAS and an artificial esophagus will further improve issues of postoperative patient complications and prognosis.

## Data Availability

Data availability is not applicable to this article as no new data were created or analyzed in this study.
